# Older Adults’ Perspectives on and Experiences with Cannabis Use: Results From the 2024 National Poll on Healthy Aging

**DOI:** 10.1093/geroni/igaf122.1969

**Published:** 2025-12-31

**Authors:** Erin Bonar, Erica Solway, Lianlian Lei, Matthias Kirch, Dianne Singer, Sydney Strunk, J Scott Roberts, Preeti Malani

**Affiliations:** University of Michigan, Ann Arbor, Michigan, United States; University of Michigan, Ann Arbor, Michigan, United States; University of Michigan, Ann Arbor, Michigan, United States; University of Michigan, Ann Arbor, Michigan, United States; University of Michigan, Ann Arbor, Michigan, United States; University of Michigan, Ann Arbor, Michigan, United States; University of Michigan School of Public Health, Ann Arbor, Michigan, United States; University of Michigan, Ann Arbor, Michigan, United States

## Abstract

Cannabis use is increasing among older adults, concurrent with regulations allowing medical and/or recreational consumption, increases in the THC potency of cannabis products, and declining public perceptions of cannabis-related risks. Using a nationally representative poll of 3,379 adults age 50 and older, we described the prevalence of cannabis use and associated characteristics (e.g., motives for consumption, cannabis use disorder [CUD] symptoms, discussions with healthcare providers). One-fifth (21%) of older adults reported past-year cannabis use (once/twice: 9%, at least monthly: 12%). Frequently endorsed motives for consumption included: relaxation (81%), sleep (68%), enhancement (i.e., enjoyment, celebration, feel good; 64%), pain (63%), and mental health/mood (53%). Among respondents who consumed cannabis at least monthly, 44% had not discussed their use with healthcare providers and many reported CUD symptoms including tolerance (17-22% across items) and craving (13%). Greater understanding of older adults’ motives for and experiences with cannabis use can guide recommendations and interventions.

